# Supplementary material for: Remodeling of lipid-foam prototissues by network-wide tension fluctuations induced by active particles
Source: Nat Commun. 2025 Feb 27;16:2026. doi: 10.1038/s41467-025-57178-x (PMC11868539; doi:10.1038/s41467-025-57178-x)
Supplement: Supplementary file 2 — Description of Additional Supplementary Information [file 41467_2025_57178_MOESM2_ESM.docx]

**Description of Additional Supplementary Files**

File Name: Supplementary Movie 1

Description: Aspiration of prototissue compartment into a micropipette at constant suction. The first arrow indicates an aspirated compartment being replaced by neighboring compartments that flow into its former position. The second arrow indicates a nearby tissue remodeling event.

File Name: Supplementary Movie 2

Description: Dynamic remodeling of prototissue after mechanical loading by a glass pipette.

File Name: Supplementary Movie 3

Description: Encapsulation of live, fluorescent *B. subtilis* bacteria (green) in prototissue compartments (boundaries displayed in red).
